# Hospital length of stay among children with and without congenital anomalies across 11 European regions—A population-based data linkage study

**DOI:** 10.1371/journal.pone.0269874

**Published:** 2022-07-22

**Authors:** Stine Kjaer Urhoj, Joachim Tan, Joan K. Morris, Joanne Given, Gianni Astolfi, Silvia Baldacci, Ingeborg Barisic, Joanna Brigden, Clara Cavero-Carbonell, Hannah Evans, Mika Gissler, Anna Heino, Sue Jordan, Renée Lutke, Ljubica Odak, Aurora Puccini, Michele Santoro, Ieuan Scanlon, Hermien E. K. de Walle, Diana Wellesley, Óscar Zurriaga, Maria Loane, Ester Garne

**Affiliations:** 1 Department of Paediatrics and Adolescent Medicine, Lillebaelt Hospital, University Hospital of Southern Denmark, Kolding, Denmark; 2 Population Health Research Institute, St George’s, University of London, London, United Kingdom; 3 Faculty of Life and Health Sciences, Ulster University, Northern Ireland, United Kingdom; 4 Dept. of Neuroscience and Rehabilitation, University of Ferrara, Ferrara, Italy; 5 Unit of Epidemiology of Rare diseases and Congenital anomalies, Institute of Clinical Physiology, National Research Council, Pisa, Italy; 6 Children’s Hospital Zagreb, Centre of Excellence for Reproductive and Regenerative Medicine, Medical School University of Zagreb, Zagreb, Croatia; 7 Rare Diseases Research Unit, Foundation for the Promotion of Health and Biomedical Research in the Valencian Region (UVEG-FISABIO), Valencia, Spain; 8 Finnish Institute for Health and Welfare, Information Services Department, Helsinki, Finland; 9 Faculty of Medicine, Health and Life Sciences, Swansea University, Wales, United Kingdom; 10 Department of Genetics, University of Groningen, University Medical Center Groningen, Groningen, The Netherlands; 11 Territorial Assistance Service–Drug and Medical Device Area, Emilia Romagna Health Department, Bologna, Italy; 12 Clinical Genetics, Princess Anne Hospital, University of Southampton and Wessex Clinical Genetics Service, Southampton, United Kingdom; 13 Department of Preventive Medicine and Public Health, Rare Diseases Research Unit UVEG-FISABIO Valencia and Spanish Consortium for Biomedical Research in Epidemiology and Public Health, University of Valencia (Spain) and Valencia Region (Spain) Health Authority (Conselleria de Sanitat Universal I Salut Pública), Valencia, Spain; Federal University of Sergipe, BRAZIL

## Abstract

**Background:**

Congenital anomalies are a leading cause of childhood morbidity, but little is known about the long-term outcomes.

**Objective:**

To quantify the burden of disease in childhood for children with congenital anomalies by assessing the risk of hospitalisation, the number of days spent in hospital and proportion of children with extended stays (≥10 days).

**Methods:**

European population-based record-linkage study in 11 regions in eight countries including children with congenital anomalies (EUROCAT children) and without congenital anomalies (reference children) living in the same regions. The children were born between 1995 and 2014 and were followed to their tenth birthday or 31/12/2015. European meta-analyses of the outcome measures were performed by two age groups, <1 year and 1–4 years.

**Results:**

99,416 EUROCAT children and 2,021,772 reference children were linked to hospital databases. Among EUROCAT children, 85% (95%-CI: 79–90%) were hospitalised in the first year and 56% (95%-CI: 51–61%) at ages 1–4 years, compared to 31% (95%-CI: 26–37%) and 25% (95%-CI: 19–31%) of the reference children. Median length of stay was 2–3 times longer for EUROCAT children in both age groups. The percentages of children with extended stays (≥10 days) in the first year were 24% (95%-CI: 20–29%) for EUROCAT children and 1% (95%-CI: 1–2%) for reference children. The median length of stay varied greatly between congenital anomaly subgroups, with children with gastrointestinal anomalies and congenital heart defects having the longest stays.

**Conclusions:**

Children with congenital anomalies were more frequently hospitalised and median length of stay was longer. The outlook improves after the first year. Parents of children with congenital anomalies should be informed about the increased hospitalisations required for their child’s care and the impact on family life and siblings, and they should be adequately supported.

## Introduction

Congenital anomalies are a leading cause of childhood morbidity and long-term disability [1], partly because the survival of children with congenital anomalies has improved [2–4]. It is therefore important to have accurate evidence- based information about the overall health of children with congenital anomalies to counsel parents after a prenatal or postnatal suspicion or diagnosis of a congenital anomaly.

Most literature to date has related to children with specific congenital anomalies identified from hospital contacts, and the overall morbidity for children with a range of congenital anomalies has not yet been published in a population-based setting. Many of the published studies consist of cohorts of live born children referred for surgery: these are biased samples as not all children are referred for surgery and they may die before surgery [5, 6]. A population-based study from Australia showed that among almost 22,000 children with major congenital anomalies born in 1980–95, the mean number of hospital admissions up to the age of five years was higher (3.8 admissions per child) than in children without congenital anomalies (2.2 admissions per child). Moreover, the mean length of stay (LOS) per year was also higher for all age groups up to 18 years [7].

The use of routinely collected health data has become an important data source for research, although they are not collected for research purposes. EUROlinkCAT is the first population-based study to link hospital admission and discharge data in several European regions to evaluate morbidity outcomes in children with congenital anomalies up to ten years of age [8].

The aim of this EUROlinkCAT paper is to quantify the burden of disease in childhood for children with congenital anomalies compared to children without congenital anomalies by assessing the risk of hospitalisation, the number of days spent in hospital and the proportion of children with extended stays.

## Material and methods

### Population

This study is a European, population-based data-linkage cohort study, including data from 11 EUROCAT registries (national and regional): Croatia, Zagreb; Denmark, Funen; Finland; Italy, Emilia Romagna and Tuscany; The Netherlands, Northern Netherlands; Spain, Valencian Region; United Kingdom, Wales, East Midlands & South Yorkshire, Thames Valley and Wessex. Live born children with major congenital anomalies as defined by EUROCAT [9, 10] and born between 1995 (or the first year of the EUROCAT registry if later) and 2014 were included (EUROCAT children).

For each of the registries, data on all live born children without congenital anomalies born during the same time-period and from the same population area covered by the registry were included as a reference population (reference children). The Tuscany and Northern Netherland registries used a random sample of 10% and a 20% of their population as the reference children (matched with EUROCAT children on sex and year of birth). For Zagreb and the three English registries (East Midlands & South Yorkshire, Thames Valley and Wessex), individual-level data on reference children were not available. Therefore, aggregate hospitalisation data published by EUROSTAT, the statistical office of the European Union [11], were used to derive comparable estimates for <1 year; see S1 Table.

As a child is only registered in a hospital database if the child has a hospital admission, children were also linked to other databases (national statistics, vital statistics, hospital databases outside study period, and hospital outpatient records) where possible to minimise the risk that missing linkage was the reason for children without any registered hospitalisations. Children who could not be linked to any of these databases were not included in the analysis (2.5% of EUROCAT children and 2.1% of reference children) [12]. Children with a date of death (age ≥1 day) and no registration of a hospital stay were excluded, as it is not known if these children died suddenly without a previous hospital contact or if the hospital contacts were not registered (5.6% of EUROCAT children and 0.1% of reference children). Details of the linkage methods used are provided in S2 Table.

### Data on hospitalisations

Data on hospitalisations for all children up to the child’s 10^th^ birthday or end of 2015 (so that all children had at least one year of follow-up), whichever came earlier, were obtained by electronic linkage to hospital databases for all registries except Zagreb. In Zagreb, hospital data for the EUROCAT children were collected manually through medical records in electronic and paper form. The Northern Netherlands used two databases, LMR (Landelijke Medische Registratie for birth years 1995–2010) and LBZ (Landelijke Basisregistratie Ziekenhuiszorg for birth years 2013–2014). Data from the LMR and LBZ database were included up to the end of 2017. Outpatient visits in the LBZ database were recorded as hospital admissions in 2013, therefore these data for reference children <1 year were dropped from the study.

The hospital databases in Finland, Denmark, the Netherlands, Italy (Tuscany), Wales and England (East Midlands & South Yorkshire, Thames Valley and Wessex) covered hospitalisations in the whole country. For Wales, this included procedures carried out in England. For Spain and Italy (Emilia Romagna), the hospital databases covered the same region as the EUROCAT registry. Data on hospitalisations between 5 and 10 years of age were available for eight of the eleven registries as only children born in 1995–2005 reached the age of 10 years before end of 2015. As the coverage in this age group was much lower, only the age groups <1 year and 1–4 years are included in the meta-analysis.

Newborns, infants and children are frequently transferred to specialist hospitals for further treatment, which are usually registered as separate hospitalisations in the hospital databases. In this study, hospital stays were counted as one stay if there was one day or less between a discharge and the next admission. LOS was calculated as the number of days between the date of admission to hospital and the date of discharge home. For hospital stays, where the date of admission and discharge occurred on the same day, the LOS was considered to be 0.5 days. If the date of discharge was after December 31^st^ 2015 or after reaching age 10 years (i.e. 3652 days after birth), the date of discharge was defined as the earliest of these dates. The date of discharge was missing for a very small number of hospital stays in six registries and we used a proxy discharge date (date of admission+(2*(‘last date’-date of admission), where ‘last date’ was the latest of the surgery and intensive care unit dates). Admissions associated with birth only (obstetric stays immediately after birth) were excluded as the majority of all children were born in a hospital and therefore will have a hospital stay. In general, admissions at date of birth (age = 0) or the day after (age = 1 day), where the only diagnosis recorded was an obstetric code i.e. ICD-10 codes Z37-Z39 (codes for the outcome of delivery) or ICD-9-CM codes V30-V39 (codes for the type of birth) were excluded. All admissions on the day of birth or on day 1 that included additional diagnosis or procedure codes were included in the study. The method of excluding obstetric stays differed between registries due to differences in the codes used for healthy newborns [8].

### Congenital anomalies

Analyses were performed on all children with major congenital anomalies (the EUROCAT subgroup “all anomalies”) and subgroups of specific congenital anomalies according to EUROCAT definitions; this included both children with an isolated anomaly and children with associated anomalies in other organ systems and/or genetic and chromosomal anomalies [8, 9]. Therefore, children with more than one major anomaly may be included in more than one congenital anomaly subgroup presented in the tables and figures. Additionally, some of the subgroups are hierarchical, for example a child with Tetralogy of Fallot will also be included in the “severe congenital heart defect (CHD)” and the “all CHD subgroups”.

### Statistical analysis

A common data model was developed for all the hospital databases which was used to standardise the variables obtained by linkage from the local databases [8]. This enabled all registries to run centrally written syntax scripts in STATA version 13 for linkage quality checks and morbidity analyses. No individual case data were shared as all analyses were performed locally using these linked datasets. The aggregate tables and analytic results produced were then sent to a Central Results Repository at Ulster University for collation and re-distribution to the study team.

All analysis were performed separately for three age groups: <1 year (0–364 days), 1–4 years (365–1825 days), and 5–9 years (1826–3652 days). The term children is used for all age groups. Meta-analyses to obtain pooled estimates of percentage hospitalised and median LOS were performed for <1 year and 1–4 years only.

In each registry for each congenital anomaly subgroup and for the reference children, the percentage of children hospitalised within each age group was calculated using Kaplan Meier (KM) survival analysis to allow for the censoring of children occurring on December 31^st^ 2015, death or emigration from the study region or country. The confidence intervals for the KM survival analysis estimates were calculated by STATA using the ln(-ln(S(t))) transformation. To obtain pooled estimates of the percentage hospitalised across registries random effects inverse-variance meta-analyses were performed using the ln(-ln(S(t))) transformation. For several anomalies where all children were hospitalised in the first year, the lower 95% confidence limit was calculated using the exact binomial estimates, the percentage of children hospitalised was estimated to be 99.9% and the upper 95% confidence limit was calculated assuming symmetry on the ln(-ln(S(t)) scale. The same method was adopted for the percentage of children who had a single hospital stay of ten days or more and this analysis was restricted to children born at term (at least 37 weeks gestation) to exclude the extremely long stays experienced by babies born preterm. Information about gestational age was not available in the Netherlands data and data for long hospital stays (≥10 days) were therefore not included. The meta-analysis was performed in Stata (version 15).

As hospital stays are not normally distributed, with a small proportion of children having very long stays, the median and interquartile range were reported for those children who had at least 1 hospital admission in that age group. In the Tuscany and Emilia Romagna registries 18% and 10% of admissions respectively, consisted of multiple separate stints in hospital with only the final discharge date being recorded, so that the days spent in hospital could not be ascertained and they were excluded from the analysis.

Quantile estimation methods were used to obtain pooled estimates of the median LOS [13]. These methods use the reported median and quartiles for each registry to select an underlying parametric distribution based on the best-fit of normal, log-normal, gamma and Weibull distributions. The asymptotic variance of the median can then be calculated and a random effects meta-analysis performed [14, 15] using the “metamedian” package in R, version 4.0.3.

Registries were not included in the meta-analyses of percentage of children hospitalised (including extended stays) or medians for a particular subgroup if there were fewer than three children. Significant outliers were queried with the registries and potential exclusion discussed based on the underlying causes. Sensitivity analyses were conducted on the pooled estimates by excluding one registry at a time and re-running the meta-analyses to identify influential outliers. Estimates outside of the original 95% confidence interval or deviations of more than 10% from the original estimate were further assessed for overall impact.

## Results

In total, 102,647 children with congenital anomalies (EUROCAT children) and 1,124,584 reference children from 11 registries in eight countries were eligible for inclusion in the study, of whom 99,416 EUROCAT children (97%) and 2,021,772 reference children (95%) were linked to hospital databases and/or vital statistics and eligible for analysis. Linkage success was very good overall, being 85–100% for all registries, except in Valencian Region where 75% of reference children were linked. In Zagreb, only EUROCAT children were linked and the linkage was manually performed; 44% of the children were linked (Table 1 and S2 Table). Table 1 shows the individual EUROCAT registries’ results for hospitalisations and LOS by age; registries varied greatly in size from over 38,000 children with congenital anomalies in Finland to 2,400 in Funen and only 380 in Zagreb. The number of reference children also varied from over 900,000 in Finland (the whole country) to 23,500 in Tuscany (a 10% sample of the population). The mean follow-up duration up to their fifth birthday was 4.2 person-years for EUROCAT children and 4.3 person-years for reference children and the mean follow-up duration in the five years from ages 5–9 was 3.8 years in EUROCAT children and 4.0 years in reference children.

**Table 1:** Number of children, percentage hospitalised and median length of stay for children with congenital anomalies and reference children by registry and age group.

		Children with CAs			Reference children		
Registry birth years	Age	Number of children^a^	Percent hospitalised^b^ (95% CI)	Median LOS^c^ (Q1;Q3)	Number of children^a^	Percent hospitalised^b^ (95% CI)	Median LOS^c^ (Q1;Q3)
**Croatia, Zagreb**^**d**^ **2008–2014**							
	<1 year	380	52.9 (48.0–58.0)	10.0 (6.0;21.0)	--	--	--
	1–4 years	376	30.6 (25.1–36.9)	2.0 (1.0;3.0)	--	--	--
	5–9 years	--	--	--	--	--	--
**Denmark, Funen 1995–2014**							
	<1 year	2,423	73.7 (71.9–75.4)	10.0 (3.0;27.0)	100,748	28.1 (27.6–28.1)	3.0 (1.0;7.0)
	1–4 years	2,285	64.8 (62.8–66.9)	1.1 (0.4;2.5)	99,945	27.6 (27.1–27.6)	0.3 (0.1;0.8)
	5–9 years	1,862	44.4 (41.9–46.9)	0.6 (0.2;1.6)	81,352	16.6 (16.0–16.6)	0.2 (0.1;0.5)
**Finland 1997–2014**							
	<1 year	38,324	60.7 (60.3–61.2)	7.0 (3.0;21.0)	911,679	21.2 (21.1–21.3)	3.0 (1.0;6.0)
	1–4 years	37,213	54.7 (54.2–55.3)	0.6 (0.3;2.0)	909,733	28.2 (28.1–28.3)	0.3 (0.1;0.6)
	5–9 years	27,121	38.8 (38.2–39.5)	0.4 (0.2;1.2)	701,127	18.0 (17.9–18.1)	0.2 (0.1;0.5)
**Italy, Emilia Romagna 2008–2014**							
	<1 year	5,381	93.9 (93.2–94.5)	8.0 (3.0;21.0)	223,995	37.3 (37.1–37.5)	3.0 (2.0;6.0)
	1–4 years	5,210	47.2 (45.6–48.8)	1.0 (0.3;3.0)	223,958	16.4 (16.3–16.6)	0.7 (0.3;1.3)
	5–9 years	--	--	--	--	--	--
**Italy, Tuscany 2005–2014**							
	<1 year	4,225	93.2 (92.4–94.0)	7.0 (3.0;21.0)	23,503	39.6 (39.0–40.3)	4.0 (3.0;6.0)
	1–4 years	4,121	49.8 (48.1–51.5)	1.0 (0.3;2.3)	23,503	18.8 (18.2–19.3)	0.5 (0.1;1.0)
	5–9 years	2,484	33.7 (31.2–36.3)	0.5 (0.2;1.8)	13,793	16.0 (15.2–17.0)	0.3 (0.1;0.8)
**The Netherlands, Northern Netherlands** ^**e**^							
LMR 1995–2010	<1 year	6,975	66.5 (65.4–67.6)	10.0 (4.0;23.0)	55,770	34.8 (34.4–35.2)	3.0 (1.0;6.0)
	1–4 years	6,520	56.4 (55.2–57.7)	0.8 (0.3;2.4)	54,770	28.6 (28.2–29.1)	0.3 (0.1;0.5)
	5–9 years	4,660	38.2 (36.8–39.7)	0.3 (0.1;0.8)	39,245	20.7 (20.3–21.1)	0.1 (0.1;0.2)
LBZ 2013–2014	<1 year	555	79.9 (76.5–83.2)	6.0 (2.5;19.0)	--	--	--
	1–4 years	530	56.4 (51.0–62.0)	0.8 (0.3;1.7)	5,730	29.3 (27.4–31.3)	0.3 (0.2;0.5)
	5–9 years	--	--	--	--	--	--
**Spain, Valencian Region 2010–2014**							
	<1 year	4,260	96.5 (95.9–97.0)	9.0 (3.0;23.0)	168,563	25.6 (25.4–25.8)	4.0 (2.0;7.0)
	1–4 years	4,093	40.9 (39.1–42.9)	2.0 (0.8;3.8)	168,495	13.3 (13.1–13.6)	0.8 (0.5;1.5)
	5–9 years	--	--	--	--	--	--
**United Kingdom, Wales 1998–2014**							
	<1 year	17,448	71.9 (71.3–72.6)	5.5 (1.5;18.0)	531,784	31.4 (31.2–31.5)	1.0 (0.5;3.5)
	1–4 years	16,558	68.5 (67.7–69.2)	0.6 (0.3;1.6)	509,565	38.0 (37.8–38.1)	0.3 (0.1;0.5)
	5–9 years	12,313	46.5 (45.5–47.5)	0.3 (0.2;0.9)	357,934	25.7 (25.5–25.9)	0.2 (0.1;.04)
**United Kingdom, East Midlands & South Yorkshire**^**d**^ **2003–2012**						<1 year: 29.7% 1–4 years: — 5–9 years: --	<1 year: 2.0% 1–4 years: — 5–9 years: --
	<1 year	11,280	91.8 (91.2–92.3)	7.0 (2.5;25.0)	--		
	1–4 years	10,210	63.8 (62.8–64.7)	0.6 (0.3;2.0)	--		
	5–9 years	7,900	46.0 (44.6–47.3)	0.3 (0.2;0.9)	--		
**United Kingdom, Thames Valley**^**d**^ **2005–2013**							
	<1 year	3,845	92.1 (91.2–93.0)	7.0 (3.0;20.5)	--		
	1–4 years	3,485	64.5 (62.7–66.2)	0.6 (0.3;1.9)	--		
	5–9 years	1,915	50.2 (46.6–54.0)	0.4 (0.2;1.0)	--		
**United Kingdom, Wessex**^**d**^ **2004–2014**							
	<1 year	4,320	89.5 (88.5–90.4)	9.0 (3.0;24.5)	--		
	1–4 years	3,955	67.4 (65.8–69.0)	0.6 (0.3;2.0)	--		
	5–9 years	2,450	50.9 (48.4–53.5)	0.5 (0.2;1.3)	--		

CA = Congenital anomalies, LOS = Length of stay, Q1 = 1^st^ quartile, Q3 = 3^rd^ quartile, — = not available / not included

^a^ Number of children at beginning of age period.

^b^ 1-Kaplan-Meier estimate of children ever hospitalised in age period.

^c^ Median LOS in days per year calculated only among children hospitalised in age period. If a hospital stay spans two age groups the remaining days will be allocated to the next age period.

^d^ Estimates for reference children <1 year for Croatia, Zagreb and the 3 English registries (East Midlands & South Yorkshire, Thames Valley and Wessex) were obtained using published healthcare activity data on EUROSTAT. For details of derivations see S1 Table.

^e^ All numbers are rounded to the nearest 5 for the 3 English registries (East Midlands & South Yorkshire, Thames Valley and Wessex) and for the Northern Netherlands. For the Northern Netherlands, two datasets, LMR and LBZ, covering the register area were used, LMR for birth years 1995–2010 and LBZ for 2013–2014. LBZ data for reference children were only included for 1–4 years as outpatient contacts in 2013 were recorded as admissions and <1 year data were therefore excluded.

The percentages of children hospitalised varied across registries, but for the majority of registries a higher percentage of children were hospitalised within the first year of life compared to the total over the next four years (ages 1–4) and the final five years (ages 5–9) for both EUROCAT children and reference children (Table 1). The annual median LOS per year was much higher in the first year than in the subsequent years for both EUROCAT children and reference children. All registries had the same pattern of much higher percentages of EUROCAT children being hospitalised than reference children and for much longer periods of time. Overall, there were no clear differences across the EUROCAT registries in median LOS for the EUROCAT children and the reference children respectively, however, the median LOS in the first year for both EUROCAT and reference children was slightly shorter in Wales; a likely contributing factor is that most children needing major surgery were transferred to England, due to a lack of specialist facilities in Wales.

Table 2 presents the meta-analyses of the percentage of children hospitalised and percentage of children with a stay of at least 10 days for reference children, all EUROCAT children and for children with specific congenital anomalies for the age groups <1 year and 1–4 years. For all EUROCAT children, the percentage hospitalised in the first year was 84.9% (95% CI: 78.6%-89.5%) compared to 31.0% (95% CI: 25.7%-36.5%) for the reference children. Lower percentages of children were hospitalised in the subsequent four years; 56.2% (51.1%-61.0%) of all EUROCAT children and 24.6% (18.8%-31.0%) of the reference children. In the first year, 23.9% (95% CI: 19.5–28.5%) of all EUROCAT children had at least one hospital stay of 10 days or more in a single admission, decreasing to 5.4% (95% CI: 4.8–6.1%) in the age group 1–4 years; the corresponding percentages were 1.2% (95%CI: 0.9%-1.7%) and 0.6% (95%CI: 0.5–0.8%) respectively in reference children. For children with some severe anomalies, including transposition of great vessels, hypoplastic left heart, oesophageal atresia, duodenal atresia or stenosis, atresia or stenosis of other parts of small intestine and gastroschisis, more than 80% of the children had hospital stays of 10 days or more in the first year.

**Table 2:** Meta-analysis of percentage hospitalised and percentage with a long stay (≥10 days) according to congenital anomaly subgroup and age.

	Children <1 year			Children 1–4 years		
	Total number^a^	Percent hospitalised^b^ (95% CI)	Percent hospitalised ≥10 days^c^ (95% CI)	Total number^a^	Percent hospitalised^b^ (95% CI)	Percent hospitalized ≥10 days^c^ (95% CI)
**Reference children** ^**d**^	2,016,042	31.0 (25.7–36.5)	1.2 (0.9–1.7)	1,995,699	24.6 (18.8–31.0)	0.6 (0.5–0.8)
**Congenital anomaly subgroup**						
All anomalies	99,414	84.9 (78.6–89.5)	23.9 (19.5–28.5)	94,555	56.2 (51.1–61.0)	5.4 (4.8–6.1)
Spina Bifida	686	95.4 (91.4–97.5)	60.1 (55.5–64.4)	592	84.3 (74.5–90.6)	17.8 (13.8–22.3)
Hydrocephalus	1,162	92.4 (89.5–94.6)	45.6 (34.5–56.0)	993	79.8 (72.4–85.4)	14.0 (11.2–17.2)
Severe microcephaly	963	87.2 (80.1–91.9)	34.5 (29.0–40.3)	866	79.7 (68.6–87.2)	15.0 (10.8–20.0)
Congenital cataract	846	82.1 (72.8–88.4)	11.7 (7.5–16.8)	810	70.8 (63.9–76.7)	4.1 (2.5–6.3)
ALL CHD	36,049	87.3 (80.3–91.9)	34.8 (26.6–43.2)	33,847	52.0 (44.1–59.2)	7.6 (5.7–9.7)
Severe CHD	8,677	94.8 (91.6–96.8)	68.7 (62.4–74.2)	7,416	66.4 (59.8–72.1)	17.1 (14.4–20.1)
Transposition of great vessels	1,239	99.2 (97.4–99.7)	85.9 (80.8–89.7)	1,071	66.9 (61.8–71.4)	15.2 (11.0–20.0)
VSD	20,296	86.7 (79.1–91.7)	29.0 (21.0–37.4)	19,475	47.3 (39.5–54.7)	6.0 (4.3–8.2)
ASD	7,072	86.4 (80.3–90.8)	37.3 (30.7–43.8)	6,698	58.0 (49.1–66.0)	7.5 (5.9–9.4)
AVSD	1,413	93.9 (89.1–96.6)	66.4 (60.5–71.7)	1,147	79.9 (73.4–84.9)	21.7 (18.8–24.8)
Tetralogy of Fallot	1,282	98.4 (95.7–99.4)	68.4 (62.1–73.8)	1,166	73.1 (65.7–79.2)	21.6 (16.8–26.8)
Pulmonary valve stenosis	2,282	81.1 (74.6–86.1)	33.5 (24.0–43.4)	2,164	56.1 (50.2–61.5)	10.9 (7.8–14.6)
Aortic valve atresia/stenosis	886	83.3 (73.8–89.6)	41.4 (30.4–52.0)	780	61.9 (52.2–70.3)	18.4 (10.0–28.8)
Mitral valve anomalies	736	85.9 (79.9–90.2)	51.8 (43.5–59.5)	632	69.4 (56.3–79.3)	25.1 (16.9–34.2)
Hypoplastic left heart	629	99.5 (98.4–99.9)	86.0 (73.8–92.7)	332	89.6 (73.3–96.2)	70.5 (46.7–85.2)
Coarctation of aorta	2,164	94.4 (90.5–96.8)	65.9 (58.7–72.1)	1,935	64.3 (56.2–71.3)	13.5 (11.6–15.5)
PDA as only CHD in term infants (> = 37 weeks)^e^	1,183	84.3 (72.7–91.3)	27.2 (19.0–36.1)	1,131	56.1 (36.2–72.0)	5.7 (2.7–10.2)
Cleft lip with or without cleft palate	3,395	97.7 (95.8–98.7)	10.4 (7.7–13.5)	3,238	62.2 (56.1–67.6)	2.2 (1.5–3.1)
Cleft palate	3,125	91.3 (85.0–95.1)	27.8 (22.6–33.3)	2,946	75.6 (67.4–82.1)	5.5 (3.7–7.7)
Esophageal atresia	1,010	98.1 (94.9–99.3)	85.0 (79.5–89.2)	891	86.7 (78.7–91.9)	16.0 (12.8–19.6)
Duodenal atresia or stenosis^f^	589	97.3 (94.3–98.8)	80.3 (68.8–88.0)	534	60.4 (49.8–69.4)	8.1 (2.5–18.1)
Atresia or stenosis other parts of small intestine	387	98.2 (96.1–99.2)	87.5 (77.5–93.2)	362	48.5 (38.4–57.8)	10.9 (4.0–21.7)
Ano-rectal atresia and stenosis	1,186	98.3 (95.9–99.3)	49.7 (40.4–58.4)	1,080	72.9 (67.2–77.8)	8.3 (6.3–10.7)
Diaphragmatic hernia	786	94.9 (90.5–97.3)	72.5 (65.2–78.4)	556	62.8 (54.2–70.2)	8.1 (5.2–11.9)
Gastroschisis	1,072	96.4 (93.3–98.1)	88.8 (81.7–93.2)	985	51.9 (44.9–58.5)	3.3 (1.3–6.9)
Omphalocele	524	93.9 (87.3–97.1)	58.4 (43.1–70.9)	421	60.1 (50.2–68.6)	8.6 (5.2–13.1)
Multicystic renal dysplasia	1,389	85.2 (77.5–90.4)	14.8 (9.0–21.9)	1,283	58.1 (51.6–64.0)	3.7 (2.5–5.2)
Congenital hydronephrosis	5,842	87.6 (82.8–91.1)	19.2 (13.2–26.2)	5,653	55.9 (49.8–61.7)	4.3 (2.5–6.7)
Hypospadias	5,960	77.3 (67.5–84.4)	8.8 (6.4–11.7)	5,763	80.8 (75.7–84.9)	5.8 (2.0–12.8)
Limb reduction defects	1,830	81.5 (73.1–87.6)	14.6 (10.9–18.8)	1,695	59.8 (54.9–64.3)	5.3 (3.6–7.5)
Clubfoot	4,645	87.8 (84.0–90.8)	8.9 (7.4–10.5)	4,409	53.2 (50.5–55.8)	3.3 (2.7–4.0)
Hip dislocation	3,449	71.6 (63.6–78.1)	13.3 (7.3–21.0)	3,369	46.0 (36.2–55.2)	7.2 (4.8–10.3)
Polydactyly	4,152	83.2 (72.5–90.0)	6.5 (4.9–8.3)	3,979	56.1 (50.2–61.7)	1.7 (1.0–2.8)
Syndactyly	2,344	77.9 (66.7–85.7)	9.0 (6.5–11.9)	2,244	65.1 (58.0–71.2)	4.5 (2.6–7.3)
Craniosynostosis	1,425	91.8 (87.0–94.8)	15.6 (10.2–22.2)	1,380	69.1 (54.7–79.7)	5.6 (4.1–7.5)
Down syndrome	4,002	91.3 (87.4–94.1)	42.0 (37.1–46.9)	3,756	74.2 (69.4–78.4)	9.3 (8.1–10.7)

a Number of children at beginning of age period. Registries with <3 cases in subgroup not included. Numbers for ‘All anomalies’ do not exactly equal numbers from Table 1 due to rounding in Table 1.

b 1-Kaplan-Meier estimate of children ever hospitalised in age period from meta-analysis of all registries, except where indicated. Registries with <3 cases in subgroup not included.

^c^ 1-Kaplan-Meier estimate of children hospitalised ≥10 days in age period from meta-analysis of all registries. Registries with <3 cases in subgroup not included. Only children born ≥37 weeks of gestation included. Information on gestational age was not available from the Northern Netherlands (LMR and LBZ) and are therefore excluded.

^d^ Data from the Northern Netherlands LBZ database not included for reference children <1 year because outpatient contacts in 2013 were recorded as admissions and <1 year data were therefore excluded.

^e^ Data from UK, Wessex not included for PDA as only CHD in term infants (<1 and 1–4 years) because case identification differed from that of other registries.

^f^ Only one register included for percent hospitalised ≥10 days, 1–4 years (see S2 Table).

Measures of heterogeneity, including the I^2^ statistic and number of registers included in each meta-analysis of percentage hospitalised can be seen in S3A and S3B Table.

**Fig 1:**
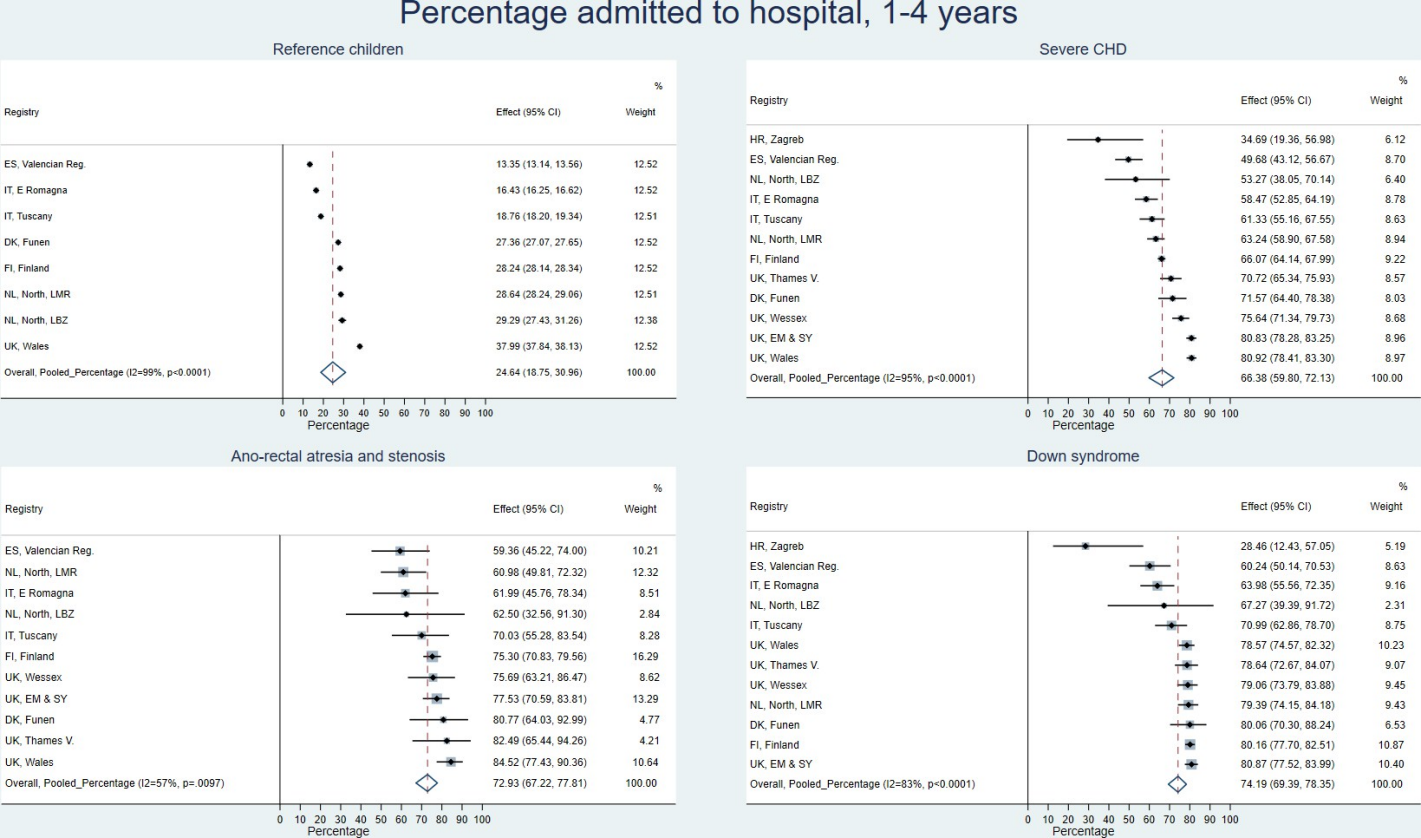
Meta-analysis of percentage hospitalised between 1–4 years for selected anomaly subgroups and reference children. Registry specific percentages (1-Kaplan-Meier estimate) of children ever hospitalised between 1–4 years and pooled percentage (based on 1-Kaplan-Meier estimates) estimated from meta-analysis of all available registers. Registries with <3 cases in subgroup not included. CHD = Congenital Heart Defects.

Fig 1 shows the estimated pooled percentage of children hospitalised as well as the registry-specific percentages hospitalised in the age group 1–4 years for the reference children and three selected anomaly subgroups: severe CHD, ano-rectal atresia and stenosis (as the most frequent gastro-intestinal anomaly) and Down syndrome. The percentage of reference children hospitalised was lower in the Valencian Region and the Italian registries (E Romagna and Tuscany) and highest in Wales. For severe CHD, the percentage ranged from 35% to 81% and the pooled estimated percentage was 66% (95% CI: 60–72%). There was a considerable degree of between-study heterogeneity, reflecting the differences in percentages hospitalised across registries (see S3A and S3B Table).

**Fig 2:**
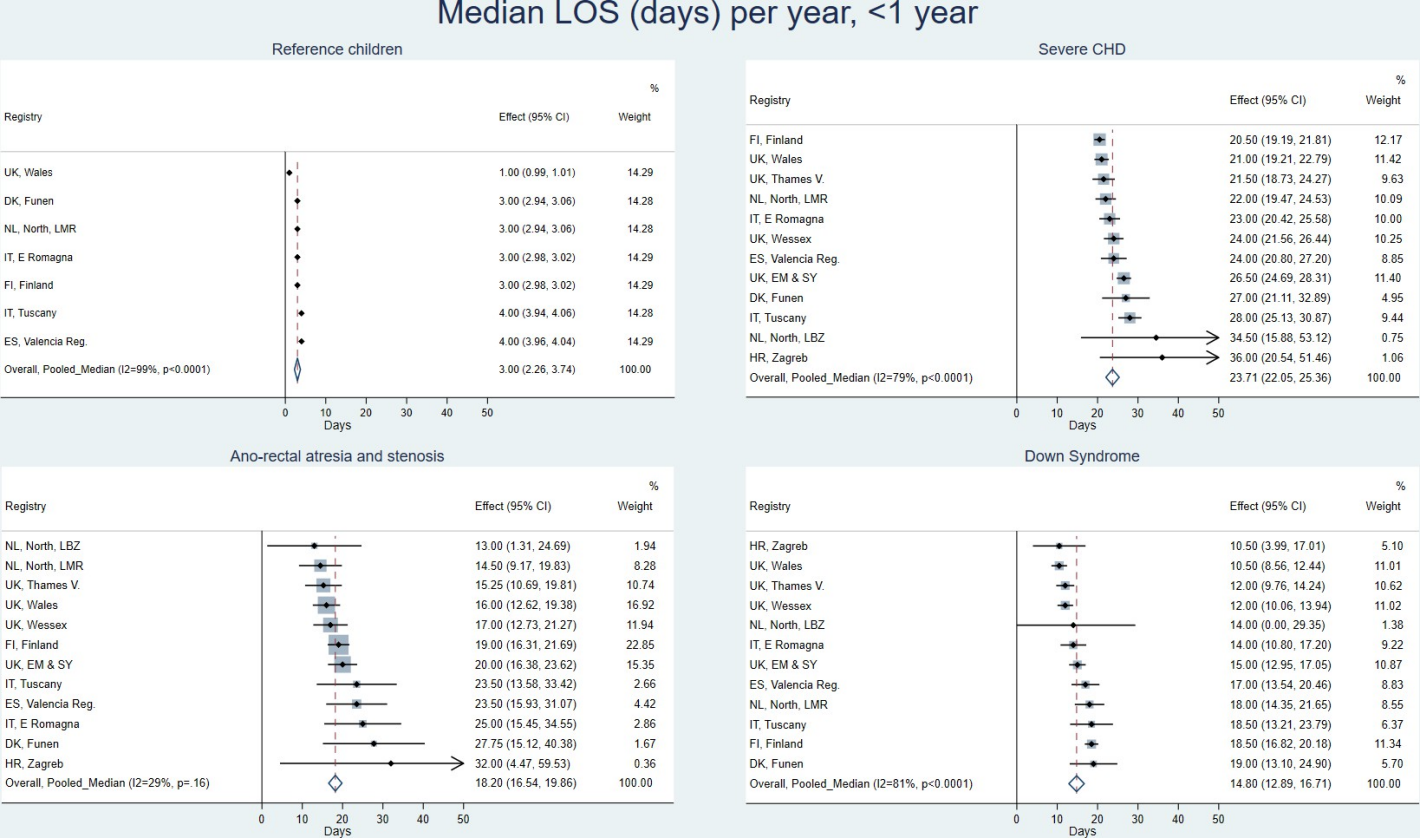
Meta-analysis of median length of stay in children <1 year for selected anomaly subgroups and reference children. Registry specific median LOS in days and pooled median LOS in days estimated from meta-analysis of all available registries. Median LOS calculated only among children hospitalised <1 year. Registries with <3 cases in subgroup not included. Data from the Northern Netherlands LBZ database not included for reference children <1 year because outpatient contacts in 2013 were recorded as admissions and <1 year data were therefore excluded. CHD = Congenital Heart Defects.

The meta-analysis of the median LOS per year for those with at least one admission by anomaly subgroup is presented in Table 3; measures of heterogeneity and number of registers included in each meta-analysis of the median LOS can be seen in S4 Table. Overall, the median LOS for the reference children was 3.0 days (95% CI: 2.3–3.7) in the first year and 0.4 days per year (95% CI: 0.2–0.5) at ages 1–4 years. The median LOS for the EUROCAT children was 7.9 days (95% CI: 7.0–8.8) in the first year and 1.0 days per year (95% CI: 0.7–1.2) at ages 1–4 years. The median LOS in the first year for children with severe CHD was 23.7 days (95% CI: 22.0–25.4). Children with gastro-intestinal anomalies, including abdominal wall defects, also had very long median LOS in the first year ranging from 18.1 days (95% CI: 14.9–21.2) for children with omphalocele to 37.4 days (95% CI: 31.3–43.5) for children with oesophageal atresia. For children with facial clefts, renal anomalies and limb anomalies the median LOS was much shorter in the first year. For children with Down syndrome, the median LOS was 14.8 days (95%CI 12.9–16.7) in the first year. The median LOS per year at ages 1–4 years was highest for children with hypoplastic left heart (4.2 days, 95% CI: 2.4–5.9). For children with hydrocephalus, AVSD, Tetralogy of Fallot and mitral valve anomalies, the median LOS was 2.0–2.3 days per year at ages 1–4 years. For all other anomaly subgroups, the median LOS was less than 2 days per year in this age group. The estimated pooled median LOS as well as the registry-specific median LOS for <1 year are presented in Fig 2 for the reference children and the three selected anomaly subgroups. Except for ano-rectal atresia and stenosis, where little inter-registry variation in median LOS is seen, there is high heterogeneity (I^2^ >75%) between registries for the remaining subgroups.

**Table 3:** Meta-analysis of median length of stay per year according to congenital anomaly subgroup and age.

	Children <1 year		Children 1–4 years	
	Total number hospitalised^a^	Median LOS^b^ (95% CI)	Total number hospitalised^a^	Median LOS^b^ (95% CI)
**Reference children** ^c^	540,046	3.0 (2.3–3.7)	498,420	0.4 (0.2–0.5)
**Congenital anomaly subgroup**				
All anomalies	73,080	7.9 (7.0–8.8)	50,829	1.0 (0.7–1.2)
Spina Bifida	634	18.2 (13.7–22.7)	502	1.8 (0.9–2.6)
Hydrocephalus	1,046	19.1 (14.4–23.7)	775	2.0 (1.5–2.5)
Severe microcephaly	787	14.8 (12.2–17.5)	632	1.9 (1.5–2.4)
Congenital cataract	630	5.1 (3.6–6.6)	533	0.6 (0.4–0.7)
ALL CHD	25,655	12.8 (10.2–15.3)	16,043	1.1 (0.9–1.3)
Severe CHD	7,905	23.7 (22.0–25.4)	4,844	1.7 (1.5–1.9)
Transposition of great vessels	1,198	24.2 (22.8–25.7)	676	1.2 (0.8–1.5)
VSD	12,952	11.5 (8.4–14.7)	7,952	1.0 (0.7–1.2)
ASD	5,461	15.4 (12.2–18.7)	3,865	1.3 (1.0–1.5)
AVSD	1,306	29.4 (26.0–32.8)	883	2.1 (1.9–2.4)
Tetralogy of Fallot	1,239	24.4 (22.1–26.7)	833	2.3 (1.8–2.9)
Pulmonary valve stenosis	1,684	12.0 (9.5–14.6)	1,109	0.9 (0.6–1.1)
Aortic valve atresia/stenosis	712	11.4 (8.1–14.7)	477	1.3 (0.7–1.9)
Mitral valve anomalies	626	19.2 (13.6–24.8)	400	2.1 (1.4–2.8)
Hypoplastic left heart^d^	545	37.0 (28.7–45.2)	302	4.2 (2.4–5.9)
Coarctation of aorta	1,975	19.4 (18.0–20.8)	1,176	1.3 (1.0–1.6)
PDA as only CHD in term infants (> = 37 weeks)^e^	995	7.0 (5.4–8.6)	624	0.8 (0.6–1.0)
Cleft lip with or without cleft palate	3,262	7.9 (6.8–9.1)	1,997	1.0 (0.7–1.4)
Cleft palate	2,857	8.8 (7.3–10.2)	2,184	1.0 (0.7–1.4)
Esophageal atresia	977	37.4 (31.3–43.5)	767	1.7 (1.4–1.9)
Duodenal atresia or stenosis	568	25.5 (20.8–30.1)	332	0.6 (0.5–0.8)
Atresia or stenosis other parts of small intestine	380	33.3 (26.4–40.2)	175	0.5 (0.4–0.7)
Ano-rectal atresia and stenosis	1,114	18.2 (16.5–19.9)	751	1.6 (1.1–2.1)
Diaphragmatic hernia	701	21.7 (18.9–24.5)	340	0.8 (0.7–1.0)
Gastroschisis^f^	1,006	34.7 (29.1–40.3)	496	0.5 (0.4–0.5)
Omphalocele	480	18.1 (14.9–21.2)	253	1.0 (0.7–1.3)
Multicystic renal dysplasia	1,080	4.6 (3.3–5.8)	716	0.7 (0.5–0.8)
Congenital hydronephrosis	4,539	7.3 (5.0–9.5)	3,017	0.9 (0.5–1.3)
Hypospadias	4,205	4.9 (3.7–6.1)	4,352	1.3 (0.9–1.8)
Limb reduction defects	1,287	5.2 (4.3–6.1)	979	0.8 (0.7–1.0)
Clubfoot	3,838	3.8 (3.3–4.3)	2,175	0.5 (0.4–0.6)
Hip dislocation	2,067	4.8 (3.7–6.0)	1,555	0.8 (0.5–1.0)
Polydactyly	2,834	2.6 (2.0–3.1)	2,134	0.4 (0.3–0.6)
Syndactyly	1,455	4.1 (3.1–5.1)	1,326	0.6 (0.5–0.7)
Craniosynostosis	1,270	7.5 (5.8–9.2)	913	1.6 (1.1–2.0)
Down syndrome	3,479	14.8 (12.9–16.7)	2,694	1.0 (0.8–1.3)

LOS = Length of stay

^a^ Number of children ever hospitalised in age period.

^b^ Median LOS in days per year. Calculated only among children hospitalised in age period. Estimated from meta-analysis of all registries, except where indicated. Registries with <3 cases in subgroup not included.

^c^ Data from the Northern Netherlands LBZ database not included for reference children <1 year because outpatient contacts in 2013 were recorded as admissions and <1 year data were therefore excluded.

^d^ Data from Denmark, Funen and the Northern Netherlands, LMR, not included for hypoplastic left heart for <1 year as they had significantly lower medians due to the lack of prenatal screening in the beginning of the period and the post birth clinical decision not to offer treatment.

^e^ Data from UK, Wessex not included for PDA as only CHD in term infants (<1 year and 1–4 years) because case identification differed from that of other registries.

^f^ Data from the Northern Netherlands, LBZ not included for gastroschisis due to small numbers and was a significant outlier.

S5 Table gives the proportions of children with each anomaly who have an isolated anomaly and their hospitalisations and median LOS. The same patterns are seen in children with isolated anomalies as in Tables 2 and 3, and for the majority of anomalies children with isolated anomalies spend around 1 day less in hospital in their first year of life. The main exception was for children with AVSD, where children with an isolated AVSD spent 18.5 days compared to 29.4 days for all children with an AVSD. After the first year of life the differences are much smaller at around 0.2 days less for most isolated anomalies.

Sensitivity analyses showed that the pooled estimates were mostly robust to the exclusion of data from individual registries, except for instances where one or two outliers significantly influenced the overall result: Wessex was excluded from all meta-analyses of patent ductus arteriosus (PDA) (all ages); for the meta-analysis of median LOS, Funen and the Northern Netherlands, LMR were excluded from hypoplastic left heart (<1 year), and the Northern Netherlands LBZ was excluded from gastroschisis (<1 year). Children with PDA in Wessex had very long stays as the EUROCAT registry only included the PDA diagnosis for children with other major non-cardiac anomalies. The median LOS <1 year was very short for children with hypoplastic left heart in the Northern Netherlands, LBZ and in Funen. This is explained by the lack of prenatal screening in these regions in the beginning of the study period and the clinical decision after birth not to offer surgical treatment. In the Northern Netherlands, LBZ there were very few children with gastroschisis and the median LOS <1 year was very long.

## Discussion

This European multi-centre study with population-based data showed that children with congenital anomalies were more often hospitalised than children without congenital anomalies with 85% being in hospital in the first year and 56% at age 1–4 years compared to 31% and 25% respectively. Further, median LOS was 2–3 times longer for both age groups for children with congenital anomalies and almost 1 out of 4 children (24%) with congenital anomalies had hospital stays of 10 or more days in the first year compared to 1 out of 100 (1%) for children without congenital anomalies.

For reference children, the percentage hospitalised and the median LOS were relatively similar in magnitude across Europe. However, for EUROCAT children there were much greater differences; the percentage hospitalized during the first year varied from 52.9% to 96.5%. The relative homogeneity for the reference children indicates that sources of variation arising from data quality issues and data processing artefacts are likely to be smaller than real clinical differences such as healthcare practices, including referral patterns, and disease-severity which may partly be related to differences in terminations of pregnancy and differences in the EUROCAT registries in the inclusion of children with less severe anomalies. The results of our meta-analyses show that geographical differences exist and summarize the extent of the differences in outcomes of children with congenital anomalies and those in the background population.

Our results are in line with the study from Australia for the birth years 1980–99 where the mean annual LOS for children with congenital anomalies was twice as high as for children without congenital anomalies up to the age of 18 years [7]. In that study, 4.6% of the live born children had a congenital anomaly and they comprised 12.0% of all hospital admissions.

We are not aware of studies reporting population-based data on median LOS in the first year or later for children with CHD. There are several published studies reporting LOS after cardiac surgery. For well-defined cardiac defects such as Tetralogy of Fallot and transposition of great arteries median LOS after surgery are reported to be 7–8 days and 17 days, respectively [16, 17]. Most of these infants have also been hospitalised before the surgery and some are hospitalised later due to complications related to the surgery, unrelated infections or other health problems. In our study, we found the median LOS in the first year for children with Tetralogy of Fallot and transposition of great arteries to be 24.4 days and 24.2 days respectively and 68.4% and 85.9% of these children respectively had at least one hospital stay of more than 10 days within the first year. Our observed total days in hospital per year appear much greater, but as our measurements include pre-surgery stays and subsequent re-admissions within the same year, we believe that they are consistent with the reported stays in the other studies. Atrial septal defect (ASD) is considered a simple CHD and not all children need surgical treatment. Median LOS after surgery is reported to be 5.6 days in two studies [18, 19]. In our study, median LOS for all children with ASD was 15.4 days in the first year and 1.3 days per year at age 1–4 years, again with the higher values reflecting the inclusion of all admissions, including pre-surgery stays and subsequent re-admissions.

Our study showed a median LOS in the first year for children with diaphragmatic hernia, oesophageal atresia and gastroschisis of 21.7, 37.4 and 34.7 days, respectively. An American study found a median LOS after surgical repair (<28 days after birth) of 30, 29 and 36 days respectively [5]. A study from Finland showed that children with gastroschisis and omphalocele spent three and almost six times as many days respectively in hospital after the initial stay compared to the background population of children within a median follow-up period of eight and ten years [20].

For children with Down syndrome, we also found a high median LOS in the first year of 14.8 days and 91.3% of all children with Down syndrome were hospitalised in the first year. As approximately half of the children with Down syndrome have additional major anomalies [21], this means that children with Down syndrome without additional major anomalies are likely to be hospitalised within the first year. A study from Australia found that children with Down syndrome were 5.2 times more in hospital than reference children and that 30% of the stays were of more than 7 days [22].

We did not look at the reason for the hospitalizations in this study and thus hospitalizations that are not related to the congenital anomaly are also included. The inclusion of the reference children, however, allows the additional hospital stays related to the congenital anomaly to be estimated. We have shown that children with congenital anomalies in general and those with specific anomalies have far longer hospital stays than children without congenital anomalies and this cannot only be explained by the surgeries needed for their congenital anomaly. For most of the specific congenital anomalies included in this study surgery takes place within the first year after birth, although for children with a cleft palate or hypospadias corrective surgery may take place later [23–26]. Despite that, children with congenital anomalies are also more often hospitalised at ages 1–4 years and median LOS is longer compared to reference children. The hospital stays may be due to medical treatment and examinations related to the congenital anomaly or it may be due to infections, accidents or in other ways unrelated to the anomaly.

The main strength of this study is the population-based setting covering all children and not only those referred to tertiary hospitals for treatment. In addition, the EUROCAT registries have high levels of case ascertainment and use standardised definitions and coding of congenital anomalies to ensure consistency across Europe. The use of reference children for comparison enables interpretation of the results for children with congenital anomalies in the context of results for unaffected children. The use of reference children will also adjust for trends over time and step-changes within health records systems. Data linkage between congenital anomaly registries and hospital databases gives a more precise estimate of the number of days in hospital because all hospital admissions are included, thus, not only admissions with a congenital anomaly discharge diagnosis are included but also hospitalisations due to, for example, an infection [7]. Another strength of the study is the use of a common data model, enabling standardised scripts to efficiently and consistently analyse data from all linked datasets. This has resulted in the creation of a large, standardised cohort across Europe, allowing data from different hospital database systems to be compared across regions and pooled to generate European results.

The major limitation of the study is that it relies on data from administrative hospital databases not collected for research purposes. Another limitation of the study is that not all live births in EUROCAT were linked to health care records. Also, 0.1% of children with a date of death before the end of study but with no registration of a hospital stay were excluded, as it is not known if these children died suddenly without a previous hospital contact or if the hospital contact was not registered. Children with severe and complex congenital anomalies may have been referred for specialised surgical or medical treatment outside the registry area before or after birth and may underestimate the morbidity. The registration of obstetric stays for newborns differs across regions as well as hospitals, and the exclusion of these stays may have introduced additional variation across registries. Lastly, the results may not be completely comparable due to different years of coverage for each registry, although there is still sufficient overlap in the years covered for results to be representative.

## Conclusion and relevance for clinicians and policy makers

Our study showed that 17 out of 20 children (85%) with major congenital anomalies were hospitalised in the first year decreasing to 11 out of 20 children (56%) at age 1–4 years. Further, the median LOS was 2–3 times longer for both age groups for children with congenital anomalies compared with children without congenital anomalies and almost 1 out of 4 children (24%) with congenital anomalies had hospital stays of 10 days or more in the first year compared to 1 out of 100 (1%) for children without congenital anomalies. Further our study gives this information on hospitalisations and LOS for 36 specific congenital anomalies.

Parents of children with congenital anomalies should be informed about the increased likelihood of lengthy hospitalisations with their child, particularly in the first year of the child’s life, and the associated challenges it creates for having a normal family life and taking care of siblings. The outlook is more positive beyond the first year, though the hospitalisations still exceed those of children without congenital anomalies. Therefore, these families should be adequately supported, not only by health care professionals, but also by relevant authorities and by the health and social policies.

## Supporting information

S1 Table Estimates of proportion of reference children admitted to hospital and length of stay (LOS) for Croatia and United Kingdom. Proportion of children admitted to hospital and length of stay by age and region.
(PDF)

S2 Table Linkage quality. Number of children and proportions linked to national/vital statistics or hospital databases.
(PDF)

S3 Table Measures of heterogeneity in meta-analysis. Percentage of children ever hospitalized and percentage of children with long hospital stays (≥10 days).
(PDF)

S4 Table Measures of heterogeneity in meta-analysis. Median length of stay per year.
(PDF)

S5 Table Isolated congenital anomalies. Tables 2 and 3 for children with isolated anomalies only.
(PDF)
