# Supplementary material for: Hospital length of stay among children with and without congenital anomalies across 11 European regions—A population-based data linkage study
Source: PLoS One. 2022 Jul 22;17(7):e0269874. doi: 10.1371/journal.pone.0269874 (PMC9307180; doi:10.1371/journal.pone.0269874)
Supplement: S1 Table — Proportion of children admitted to hospital and length of stay by age and region. (PDF) [file pone.0269874.s001.pdf]

Appendix 1

Estimates of proportion of reference children admitted to hospital and length of stay (LOS) for Croatia and United Kingdom

As individual-level hospitalization data for children in the background population were not available for the Zagreb register and the three English registries (East Midlands & South Yorkshire, Thames Valley and Wessex), estimates were obtained using published healthcare activity data on EUROSTAT (https://ec.europa.eu/eurostat/web/main/data/database; accessed 01/08/2021). Source datasets used included total hospital discharges, discharges per 100,000 inhabitants and total hospital bed days, for national and selected regional datasets corresponding to the EUROCAT registry catchment areas. Data from 2005–2015 were extracted for discharges with all ICD-10 diagnosis codes except V00-Y98 (external causes) and Z38 (livebirth), so that it would be broadly comparable to our study data. For simplicity, we equated the number of discharges with the number of admissions. For each data year, age group and region, the total number of inhabitants was calculated from the number of admissions and the admission rate (per 100,000). From this, the average number of admissions per 100 inhabitants over the whole period was calculated. Similarly, the mean length of stay (days) per admission was calculated by dividing the total bed days over the total number of admissions. The results from EUROSTAT are compared against the reference children values of the corresponding registry (where available) in Table S1 below. The estimation for reference children for Croatia and United Kingdom derived from EUROSTAT data is explained below the table.

Table S1: Proportion of children admitted to hospital and length of stay by age and region

EUROSTAT country/ region	Comparable EUROlinkCAT registry	Age group	EUROSTAT admissions per 100 inhabitants, in a year (A)	EUROlinkCAT % reference children with ≥1 admission (B)	EUROSTAT Mean days per admission (C)	EUROlinkCAT Reference children Median LOS (D)
Croatia	Zagreb	<1 year	52.2	--	8.1	--
		1-4 years	12.0	--	5.6	--
		5-9 years	8.3	--	5.8	--
Denmark	Funen	<1 year	38.9	28.1	6.8	3.0
		1-4 years	8.5	27.6	2.7	0.3
		5-9 years	4.1	16.6	3.1	0.2
Finland	Finland	<1 year	31.5	21.2	6.2	3.0
		1-4 years	6.7	28.2	2.8	0.3
		5-9 years	3.7	18.0	5.5	0.2

Emilia-Romagna	Emilia-Romagna	<1 year	77.2	37.3	7.5	3.0
		1-4 years	15.5	16.4	3.8	0.7
		5-9 years	9.7	--	3.6	--
Tuscany	Tuscany	<1 year	22.8	39.6	7.7	4.0
		1-4 years	6.3	18.8	4.4	0.5
		5-9 years	3.8	--	4.1	--
Netherlands	North Netherlands, LMR	<1 year	61.4	34.8	5.5	3.0
		1-4 years	5.2	28.6	3.8	0.3
		5-9 years	2.2	20.7	3.6	0.1
Valencian Region	Valencian Region	<1 year	33.8	25.6	6.7	4.0
		1-4 years	6.9	13.3	3.4	0.8
		5-9 years	3.5	--	3.2	--
United Kingdom	Wales	<1 year	48.5	31.4	5.1	1.0
		1-4 years	7.0	38.0	2.5	0.3
		5-9 years	3.5	25.7	2.6	0.2
United Kingdom	East Midlands & South Yorkshire	<1 year	48.5	--	5.1	--
	Thames Valley	1-4 years	7.0	--	2.5	--
	Wessex	5-9 years	3.5	--	2.6	--

-- Not available
Hospitalization discharge data from EUROSTAT, 2005 – 2015; includes diagnosis ICD-10 codes "All causes of diseases (A00-Z99) excluding V00-Y98 and Z38".

Estimation of proportion of children admitted to hospital: admissions per 100 inhabitants (A) and proportion of children with 1 or more admissions (B) in each age group can be approximated by a linear relationship for <1 year. Excluding Tuscany as an outlier (where there were fewer admissions than children admitted), a fitted regression line estimates B=14.32 + 0.32A, which translates to 30.9% and 29.7% of reference children admitted to hospital in the first year for Croatia and United Kingdom respectively. For ages 1-4 years and 5-9 years, there were no discernible trends due to differences in the quantities measured (i.e. mean number of admissions per year vs. number of children with one or more admissions within the age intervals) and thus proportions admitted for 1-4 years and 5-9 years were not calculated.

Length of stay (LOS): the study median LOS (D) appears relatively robust to variations in the EUROSTAT mean (C). This is within expected given that LOS is right-skewed, with a few children having very long stays. A linear regression model was used to predict the median using the mean; this was only statistically significant for the median LOS at <1 year and results are shown in Table1; the EUROSTAT data were not used to estimate LOS for 1-4 years or 5-9 years. It should be noted that their purpose is indicative only as it is not possible to derive precise estimates without further modelling assumptions or additional data. The estimates for the reference children in our study are consistent with published statistics in EUROSTAT [12] for <1 year and hence the use of the EUROSTAT estimates for reference children <1 year for Croatia, Zagreb and the 3 English registries (East Midlands & South Yorkshire, Thames Valley and Wessex) is reasonable.
