# Supplementary material for: Hospital length of stay among children with and without congenital anomalies across 11 European regions—A population-based data linkage study
Source: PLoS One. 2022 Jul 22;17(7):e0269874. doi: 10.1371/journal.pone.0269874 (PMC9307180; doi:10.1371/journal.pone.0269874)
Supplement: S2 Table — Number of children and proportions linked to national/vital statistics or hospital databases. (PDF) [file pone.0269874.s002.pdf]

Appendix 2

Table S2. Linkage quality. Number of children and proportions linked to national/vital statistics or hospital databases

	Live born children ≥23 weeks gestation (n)		Children linked to national/vital statistics or hospital databases[1] (%)	
Registry	EUROCAT children	Reference children	EUROCAT children	Reference children
Croatia, Zagreb	868	--	43.8	--
Denmark, Funen	2,423	100,785	99.9	100
Finland	38,555	914,150	99.4	99.7
Italy, Emilia Romagna	5,801	263,574	92.8	85.0
Italy, Tuscany	4,821	23,789	87.6	98.8
N Netherlands, LMR (1995-2010) [2]	7,315	57,555	95.4	96.9
N Netherlands, LBZ (2013-2014) [2]	585	6,055	94.9	94.8
Spain, Valencian Region	4,308	223,760	98.9	75.3
UK, Wales	17,641	532,592	98.9	99.8
UK, East Midlands & South Yorkshire	11,605	--	97.2	--
UK, Thames Valley	3990	--	96.4	--
UK, Wessex	4,735	--	91.2	--

-- Not available

[1] Proportion of children linked to national/vital statistics and/or a hospital database at any point in time (including records outside the study period and outpatient/emergency records).

[2] Numbers rounded to nearest 5 to avoid possible disclosure due to rule of Dutch statistics (N Netherlands).
