# Supplementary material for: Hospital length of stay among children with and without congenital anomalies across 11 European regions—A population-based data linkage study
Source: PLoS One. 2022 Jul 22;17(7):e0269874. doi: 10.1371/journal.pone.0269874 (PMC9307180; doi:10.1371/journal.pone.0269874)
Supplement: S3 Table — Percentage of children ever hospitalized and percentage of children with long hospital stays (≥10 days). (PDF) [file pone.0269874.s003.pdf]

Appendix 3

Table S3a. Measures of heterogeneity and number of registries included in meta-analysis of percentage ever hospitalized (Table 2) according to anomaly subgroup and age

	% admitted, <1 year			% admitted, 1-4 years		
	I^2	P-value[a]	N registries[b]	I^2	P-value[a]	N registries[b]
**Reference children[c]**	99.98	<0.001	7	99.98	<0.001	8
**Congenital anomaly subgroup**						
All anomalies	99.84	<0.001	12	99.39	<0.001	12
Spina Bifida	38.76	0.091	11	70.99	<0.001	11
Hydrocephalus	44.25	0.056	11	75.60	<0.001	10
Severe microcephaly	78.81	<0.001	11	84.14	<0.001	11
Congenital cataract	76.43	<0.001	11	53.40	0.023	10
ALL CHD	99.65	<0.001	12	99.19	<0.001	12
Severe CHD	96.24	<0.001	12	95.06	<0.001	12
Transposition of great vessels	75.59	<0.001	11	51.88	0.028	10
VSD	99.43	<0.001	12	98.42	<0.001	12
ASD	97.29	<0.001	12	96.55	<0.001	12
AVSD	77.52	<0.001	12	68.24	<0.001	12
Tetralogy of Fallot	78.32	<0.001	10	79.71	<0.001	10
Pulmonary valve stenosis	88.57	<0.001	12	77.83	<0.001	10
Aortic valve atresia/stenosis	76.28	<0.001	11	71.43	<0.001	9
Mitral valve anomalies	41.69	0.080	10	79.37	<0.001	9
Hypoplastic left heart	20.00	0.259	10	75.88	<0.001	8
Coarctation of aorta	83.56	<0.001	11	86.40	<0.001	10
PDA as only CHD in term infants (>=37 weeks)[d]	90.41	<0.001	10	95.96	<0.001	9
Cleft lip with or without cleft palate	81.02	<0.001	12	87.56	<0.001	12
Cleft palate	93.31	<0.001	12	91.86	<0.001	11
Oesophageal atresia	63.80	0.001	12	81.51	<0.001	10
Duodenal atresia or stenosis	31.22	0.150	11	71.99	<0.001	9
Atresia or stenosis other parts of small intestine	0.00	0.639	10	59.48	0.008	10

Ano-rectal atresia and stenosis	68.24	<0.001	12	57.08	0.010	11
Diaphragmatic hernia	55.34	0.017	10	62.62	0.004	10
Gastroschisis	44.43	0.055	11	58.95	0.007	11
Omphalocele	58.16	0.008	11	58.81	0.013	9
Multicystic renal dysplasia	85.50	<0.001	11	68.14	<0.001	11
Congenital hydronephrosis	95.91	<0.001	12	92.11	<0.001	12
Hypospadias	98.32	<0.001	12	92.95	<0.001	12
Limb reduction defects	93.06	<0.001	11	61.82	0.004	11
Clubfoot	90.71	<0.001	11	47.32	0.041	11
Hip dislocation	94.21	<0.001	10	94.83	<0.001	10
Polydactyly	97.97	<0.001	11	88.05	<0.001	11
Syndactyly	96.29	<0.001	11	83.51	<0.001	11
Craniosynostosis	77.67	<0.001	11	93.30	<0.001	10
Down syndrome	92.01	<0.001	12	83.02	<0.001	12

[a] p-value for heterogeneity (Cochran's Q)

[b] Number of registries included in meta-analysis. Registries with <3 cases in subgroup not included.

[c] Data from the Northern Netherlands LBZ database not included for reference children <1 year because outpatient contacts in 2013 were recorded as admissions and <1 year data were therefore excluded.

[d] Data from UK, Wessex not included for PDA as only CHD in term infants (<1 year and 1-4 years) because case identification differed from that of other registries.

Table S3b. Measures of heterogeneity and number of registries included in meta-analysis of percentage hospitalized ≥10 days (Table 2) according to anomaly subgroup

	% ≥10 days, <1 year			% ≥10 days, 1-4 years		
	I^2	P-value[a]	N registries[b]	I^2	P-value[a]	N registries[b]
**Reference children**	99.80	<0.001	6	98.89	<0.001	6
**Congenital anomaly subgroup**						
All anomalies	99.43	<0.001	10	89.16	<0.001	10
Spina Bifida	0.00	0.573	10	0.00	0.443	3
Hydrocephalus	86.01	<0.001	9	3.21	0.405	8
Severe microcephaly	39.92	0.101	9	36.13	0.153	7
Congenital cataract	52.73	0.039	8	0.00	0.754	2
ALL CHD	99.39	<0.001	10	95.62	<0.001	10
Severe CHD	95.38	<0.001	10	80.59	<0.001	10
Transposition of great vessels	59.91	0.011	9	56.24	0.025	8
VSD	98.88	<0.001	10	92.39	<0.001	10
ASD	94.31	<0.001	10	67.87	0.002	9
AVSD	59.59	0.008	10	0.00	0.805	9
Tetralogy of Fallot	66.24	0.003	9	57.99	0.015	9
Pulmonary valve stenosis	92.19	<0.001	9	60.18	0.014	8
Aortic valve atresia/stenosis	77.48	<0.001	9	74.62	0.001	6
Mitral valve anomalies	44.09	0.085	8	54.50	0.052	6
Hypoplastic left heart	59.87	0.011	9	84.24	<0.001	6
Coarctation of aorta	82.40	<0.001	9	0.00	0.637	7
PDA as only CHD in term infants (>=37 weeks)[c]	87.09	<0.001	8	75.83	0.016	3
Cleft lip with or without cleft palate	78.87	<0.001	10	18.26	0.298	5
Cleft palate	83.53	<0.001	10	60.05	0.010	9
Oesophageal atresia	43.35	0.069	10	0.00	0.873	8
Duodenal atresia or stenosis	51.13	0.037	9	0.00	--	1
Atresia or stenosis other parts of small intestine	32.88	0.166	8	0.00	0.346	2
Ano-rectal atresia and stenosis	77.72	<0.001	10	0.00	0.518	6
Diaphragmatic hernia	39.16	0.107	9	0.00	0.549	4

Gastroschisis	53.78	0.034	8	0.00	0.347	2
Omphalocele	71.69	<0.001	9	0.00	0.422	4
Multicystic renal dysplasia	84.36	<0.001	9	0.00	0.733	5
Congenital hydronephrosis	96.06	<0.001	10	86.84	<0.001	8
Hypospadias	87.23	<0.001	10	97.29	<0.001	9
Limb reduction defects	69.03	0.001	9	36.81	0.161	6
Clubfoot	55.84	0.027	8	0.00	0.966	7
Hip dislocation	92.52	<0.001	8	66.02	0.086	2
Polydactyly	63.70	0.005	9	51.48	0.103	4
Syndactyly	60.09	0.010	9	62.88	0.019	6
Craniosynostosis	80.69	<0.001	9	7.47	0.356	4
Down syndrome	81.92	<0.001	10	13.31	0.323	9

-- = not available.

[a] p-value for heterogeneity (Cochran's Q).

[b] Number of registries included in meta-analysis. Registries with <3 cases in subgroup not included. Data on hospitalization ≥10 days not available from the Netherlands as information on gestational age was not available from the Northern Netherlands (LMR and LBZ).

[c] Data from UK, Wessex not included for PDA as only CHD in term infants (<1 year and 1-4 years) because case identification differed from other registries.
