# Supplementary material for: Hospital length of stay among children with and without congenital anomalies across 11 European regions—A population-based data linkage study
Source: PLoS One. 2022 Jul 22;17(7):e0269874. doi: 10.1371/journal.pone.0269874 (PMC9307180; doi:10.1371/journal.pone.0269874)
Supplement: S4 Table — Median length of stay per year. (PDF) [file pone.0269874.s004.pdf]

Appendix 4

Table S4. Measures of heterogeneity and number of registries included in meta-analysis of median length of stay per year (Table 3) according to anomaly subgroup

	Children <1 year			Children 1-4 years		
	I^2	P-value[a]	N registries[b]	I^2	P-value[a]	N registries[b]
**Reference children[c]**	99.99	<0.001	7	99.94	<0.001	8
**Congenital anomaly subgroup**						
All anomalies	97.98	<0.001	12	98.26	<0.001	12
Spina Bifida	85.82	<0.001	11	83.74	<0.001	11
Hydrocephalus	73.86	<0.001	11	65.35	0.001	11
Severe microcephaly	36.13	0.110	11	0.00	0.605	11
Congenital cataract	68.89	<0.001	11	56.96	0.013	10
ALL CHD	97.65	<0.001	12	96.28	<0.001	12
Severe CHD	79.40	<0.001	12	68.68	<0.001	12
Transposition of great vessels	38.88	0.090	11	65.42	0.002	10
VSD	97.47	<0.001	12	93.84	<0.001	12
ASD	91.70	<0.001	12	85.64	<0.001	12
AVSD	49.41	0.026	12	0.00	0.662	12
Tetralogy of Fallot	38.98	0.098	10	76.33	<0.001	10
Pulmonary valve stenosis	77.13	<0.001	12	70.97	<0.001	10
Aortic valve atresia/stenosis	67.07	0.001	11	78.80	<0.001	9
Mitral valve anomalies	62.07	0.005	10	49.10	0.047	9
Hypoplastic left heart[d]	70.90	0.001	8	89.47	<0.001	8
Coarctation of aorta	11.76	0.332	11	56.41	0.014	10
PDA as only CHD in term infants (>=37 weeks)[e]	66.93	0.001	10	61.29	0.006	10
Cleft lip with or without cleft palate	92.79	<0.001	12	96.09	<0.001	12
Cleft palate	77.44	<0.001	12	92.91	<0.001	12
Oesophageal atresia	64.40	0.001	12	0.00	0.457	11
Duodenal atresia or stenosis	68.23	<0.001	11	36.06	0.130	9
Atresia or stenosis other parts of small intestine	48.49	0.042	10	34.29	0.134	10

Ano-rectal atresia and stenosis	29.14	0.160	12	67.33	0.001	11
Diaphragmatic hernia	50.30	0.034	10	39.59	0.094	10
Gastroschisis[f]	81.96	<0.001	10	46.21	0.053	10
Omphalocele	59.29	0.006	11	0.00	0.502	9
Multicystic renal dysplasia	81.45	<0.001	11	68.42	<0.001	11
Congenital hydronephrosis	96.95	<0.001	12	92.43	<0.001	12
Hypospadias	93.56	<0.001	12	99.24	<0.001	12
Limb reduction defects	53.52	0.018	11	70.88	<0.001	11
Clubfoot	87.10	<0.001	11	84.94	<0.001	11
Hip dislocation	78.60	<0.001	10	81.97	<0.001	10
Polydactyly	94.31	<0.001	11	84.22	<0.001	11
Syndactyly	88.40	<0.001	11	61.90	0.003	11
Craniosynostosis	83.19	<0.001	11	93.37	<0.001	10
Down syndrome	81.99	<0.001	12	77.55	<0.001	12

[a] p-value for heterogeneity (Cochran's Q).

[b] Number of registries included in meta-analysis. Registries with <3 cases in subgroup not included.

[c] Data from the Northern Netherlands LBZ database not included for reference children <1 year because outpatient contacts in 2013 were recorded as admissions and <1 year data were therefore excluded.

[d] Data from Denmark, Funen and the Northern Netherlands, LMR, not included for hypoplastic left heart for <1 year as they had significantly lower medians due to the lack of prenatal screening in the beginning of the period and the post birth clinical decision not to offer treatment.

[e] Data from UK, Wessex not included for PDA as the only CHD in term infants (<1 year and 1-4 years) because case identification differed from that of other registries.

[f] Data from Netherlands, North, LBZ not included for gastroschisis <1 year due to small numbers and was a significant outlier.
