# Supplementary material for: Hospital length of stay among children with and without congenital anomalies across 11 European regions—A population-based data linkage study
Source: PLoS One. 2022 Jul 22;17(7):e0269874. doi: 10.1371/journal.pone.0269874 (PMC9307180; doi:10.1371/journal.pone.0269874)
Supplement: S5 Table — Tables 2 and 3 for children with isolated anomalies only. (PDF) [file pone.0269874.s005.pdf]

Appendix 5

Table 2 and 3 from the paper for children with isolated anomalies only.

**Table S5.1**	**Children <1 year**			**Children 1-4 years**		
**Congenital anomaly subgroup**	**Total number[a]**	**Percent hospitalised[b] (95% CI)**	**Percent hospitalised ≥10 days[c] (95% CI)**	**Total number[a]**	**Percent hospitalised[b] (95% CI)**	**Percent hospitalised ≥10 days[c] (95% CI)**
Spina Bifida	402	95.4 (90.4-97.8)	57.8 (50.2-64.8)	369	84.9 (74.9-91.2)	12.7 (7.8-18.9)
Hydrocephalus	616	90.0 (84.9-93.5)	34.5 (22.4-46.9)	558	75.9 (67.9-82.2)	7.4 (4.4-11.4)
Severe microcephaly	376	79.2 (62.8-88.9)	19.5 (12.5-27.7)	360	67.6 (48.8-80.8)	13.3 (3.9-28.3)
Congenital cataract	693	77.7 (65.8-85.9)	6.2 (2.5-12.4)	679	68.6 (61.4-74.7)	None
ALL CHD	28,091	85.0 (76.9-90.4)	28.7 (20.2-37.9)	27,027	45.3 (37.4-52.8)	5.7 (3.9-8.0)
Severe CHD	6,213	93.9 (90.1-96.3)	66.2 (58.2-73.0)	5,498	61.0 (53.6-67.5)	15.0 (12.0-18.3)
Transposition of great vessels	1,080	99.0 (97.1-99.7)	85.8 (80.6-89.7)	957	64.5 (58.9-69.5)	14.2 (10.3-18.8)
VSD	16,906	84.6 (76.0-90.3)	22.6 (15.2-30.8)	16,555	40.7 (33.3-47.9)	4.5 (3.0-6.6)
ASD	4,879	82.1 (74.4-87.7)	29.0 (22.6-35.8)	4,718	51.0 (42.0-59.3)	5.2 (3.9-6.7)
AVSD	488	87.6 (78.2-93.2)	53.1 (44.9-60.6)	426	70.9 (59.3-79.8)	21.2 (16.3-26.4)
Tetralogy of Fallot	860	98.3 (94.8-99.5)	62.2 (54.6-68.8)	817	66.1 (57.2-73.7)	16.5 (11.7-22.1)
Pulmonary valve stenosis	1,816	78.6 (71.2-84.4)	28.9 (18.2-40.5)	1,753	50.6 (44.1-56.7)	8.6 (5.7-12.3)
Aortic valve atresia/stenosis	721	81.2 (70.7-88.3)	41.6 (29.0-53.8)	652	58.8 (49.4-67.0)	18.2 (8.9-30.2)
Mitral valve anomalies	535	82.0 (73.5-88.0)	47.9 (40.2-55.1)	471	67.6 (54.4-77.8)	22.1 (10.5-36.5)
Hypoplastic left heart	521	99.5 (98.2-99.9)	87.6 (75.7-93.9)	283	90.1 (75.4-96.2)	71.8 (50.0-85.3)
Coarctation of aorta	1,676	93.5 (88.8-96.3)	64.3 (56.2-71.3)	1,557	61.1 (52.0-69.0)	12.0 (10.1-14.1)
PDA as only CHD in term infants[e]	686	81.2 (64.8-90.5)	15.0 (9.4-21.9)	656	49.2 (30.6-65.4)	2.6* (0.7-7.0)
Cleft lip with or without cleft palate	2,820	97.7 (95.7-98.7)	6.4 (4.1-9.4)	2,769	59.9 (54.1-65.3)	1.1 (0.6-1.9)
Cleft palate	2,067	89.7 (81.5-94.3)	16.4 (9.4-25.0)	2,026	71.9 (63.4-78.7)	2.1 (0.9-4.3)
Esophageal atresia	437	98.7 (95.6-99.6)	82.0 (74.9-87.3)	409	80.9 (71.0-87.7)	10.0 (5.6-16.0)
Duodenal atresia or stenosis	294	97.6 (92.1-99.3)	79.6 (60.5-90.2)	259	52.7 (42.2-62.1)	None
Atresia or stenosis small intestine	260	98.9 (96.5-99.7)	86.9 (74.4-93.5)	250	44.9 (34.3-55.0)	None
Ano-rectal atresia and stenosis	479	98.1 (94.3-99.4)	35.3 (22.9-47.9)	461	60.1 (53.2-66.3)	4.6 (2.2-8.3)
Diaphragmatic hernia	521	94.2 (90.1-96.7)	69.5 (59.1-77.8)	390	57.5 (48.9-65.2)	6.8* (2.4-14.2)

Gastroschisis	951	96.7 (93.3-98.3)	89.9 (81.7-94.5)	890	51.9 (46.0-57.5)	3.4 (1.3-7.2)
Omphalocele	246	94.6 (90.5-97.0)	51.5 (33.2-67.1)	216	48.3 (38.9-57.0)	None
Multicystic renal dysplasia	1,137	83.5 (74.5-89.6)	11.8 (5.5-20.6)	1,093	55.1 (48.0-61.7)	2.3 (1.2-4.1)
Congenital hydronephrosis	4,852	86.2 (80.8-90.2)	14.6 (9.2-21.3)	4,749	54.6 (48.2-60.6)	3.7 (1.9-6.5)
Hypospadias	4,961	74.4 (62.7-83.0)	5.1 (3.2-7.5)	4,845	79.9 (74.5-84.2)	5.7 (1.4-14.3)
Limb reduction defects	918	76.7 (63.3-85.8)	5.8 (3.2-9.7)	893	46.8 (43.2-50.3)	2.6* (0.9-5.6)
Clubfoot	3,655	86.6 (81.9-90.2)	3.2 (2.6-3.9)	3,561	47.4 (44.4-50.4)	1.0 (0.6-1.5)
Hip dislocation	2,664	65.2 (55.4-73.3)	7.7 (2.9-15.7)	2,627	39.1 (28.3-49.7)	4.9* (3.6-6.6)
Polydactyly	3,486	81.6 (69.1-89.4)	2.0 (1.3-3.1)	3,439	51.9 (45.9-57.6)	1.5 (0.7-2.8)
Syndactyly	1,611	71.5 (55.9-82.4)	2.0 (1.1-3.4)	1,580	55.0 (47.5-61.9)	1.4 (0.8-2.3)
Craniosynostosis	1,002	91.6 (85.6-95.2)	7.8 (2.4-17.6)	992	62.6 (47.0-74.8)	3.0 (1.3-5.9)

Foot notes table 2 isolated:

[a] Number of children at beginning of age period. Registries with <3 cases in subgroup not included.

[b] 1-Kaplan-Meier estimate of children ever hospitalised in age period from meta-analysis of all registries, except where indicated. Registries with <3 cases in subgroup not included.

[c] 1-Kaplan-Meier estimate of children hospitalised ≥10 days in age period from meta-analysis of all registries. Registries with <3 cases in subgroup not included. Only children born ≥37 weeks of gestation included. Information on gestational age was not available from the Northern Netherlands (LMR and LBZ) and are therefore excluded.

[d] Data from the Northern Netherlands LBZ database not included for reference children <1 year because outpatient contacts in 2013 were recorded as admissions and <1 year data were therefore excluded.

[e] Data from UK, Wessex not included for PDA as only CHD in term infants (<1 and 1-4 years) because case identification differed from that of other registries.

*Only one register included in analysis.

**Table S5.2**	Children <1 year		Children 1-4 years	
Median LOS for admitted children with isolated anomalies	Percentage of children with an isolated anomaly, %[a]	Median LOS[b] (95%CI)	Percentage of children with an isolated anomaly, %[a]	Median LOS[b] (95%CI)
Spina Bifida	60,3	18.8 (14.7-22.9)	62,4	1.5 (0.8-2.1)
Hydrocephalus	51,5	14.4 (9.6-19.3)	52,8	1.2 (0.8-1.6)
Severe microcephaly	35,1	9.5 (6.1-12.9)	34,0	0.8 (0.5-1.0)
Congenital cataract	77,0	3.9 (2.7-5.0)	79,9	0.4 (0.3-0.6)
ALL CHD	72,0	10.0 (7.8-12.2)	68,3	0.8 (0.7-1.0)
Severe CHD	70,5	21.3 (19.4-23.2)	67,7	1.4 (1.1-1.6)
Transposition of great vessels	87,4	23.6 (21.9-25.2)	85,9	0.9 (0.8-1.1)
VSD	76,5	8.6 (6.1-11.0)	72,8	0.7 (0.5-0.9)
ASD	63,3	12.4 (9.3-15.5)	60,3	1.1 (0.8-1.4)
AVSD	32,5	18.5 (13.5-23.5)	32,8	1.9 (1.4-2.4)
Tetralogy of Fallot	66,3	21.4 (18.6-24.1)	62,1	1.6 (1.1-2.1)
Pulmonary valve stenosis	75,7	9.0 (6.3-11.7)	71,6	0.6 (0.5-0.8)
Aortic valve atresia/stenosis	79,5	10.3 (7.3-13.4)	78,0	1.3 (0.6-2.1)
Mitral valve anomalies	70,0	18.7 (13.0-24.5)	70,8	1.7 (1.1-2.2)
Hypoplastic left heart	82,4	38.6 (31.0-46.2)	85,8	4.3 (2.6-6.1)
Coarctation of aorta	76,5	17.2 (14.6-19.8)	75,3	1.2 (0.9-1.5)
PDA as only CHD in term infants	52,7	3.7 (3.1-4.3)	43,8	0.5 (0.5-0.6)
Cleft lip with or without cleft palate	83,4	7.7 (6.6-8.7)	81,9	0.9 (0.6-1.2)
Cleft palate	65,2	6.5 (5.5-7.5)	64,4	0.9 (0.6-1.3)
Oesophageal atresia with or without fistula	43,4	31.4 (24.2-38.7)	43,5	1.0 (0.7-1.3)
Duodenal atresia or stenosis	49,6	22.6 (17.9-27.4)	41,6	0.3 (0.2-0.4)
Atresia or stenosis small intestine	67,6	30.5 (22.3-38.6)	64,0	0.5 (0.3-0.8)
Ano-rectal atresia and stenosis	40,9	11.8 (9.4-14.2)	35,0	0.5 (0.4-0.6)
Diaphragmatic hernia	67,5	20.3 (16.4-24.3)	64,1	0.4 (0.3-0.6)
Gastroschisis	89,5	35.3 (29.4-41.2)	88,4	0.4 (0.4-0.5)

Omphalocele	48,8	16.2 (12.4-20.1)	40,3	0.7 (0.3-1.0)
Multicystic renal dysplasia	80,2	3.8 (2.8-4.8)	80,6	0.6 (0.4-0.7)
Congenital hydronephrosis	80,2	6.1 (4.1-8.0)	79,4	0.8 (0.4-1.2)
Hypospadias	79,5	4.0 (3.1-4.9)	82,4	1.3 (0.8-1.7)
Limb reduction defects	47,2	3.1 (2.6-3.5)	39,6	0.6 (0.2-0.9)
Club foot – talipes equinovarus	77,3	3.0 (2.4-3.7)	71,3	0.4 (0.3-0.5)
Hip dislocation and/or dyspasia	72,2	3.5 (3.1-3.9)	70,2	0.5 (0.3-0.7)
Polydactyly	80,9	2.2 (1.7-2.6)	80,7	0.3 (0.2-0.5)
Syndactyly	61,4	2.7 (2.2-3.2)	60,8	0.5 (0.3-0.6)
Craniosynostosis	70,1	6.3 (4.6-8.0)	66,0	1.5 (1.0-2.1)
